# Metal - Insulator Transition Driven by Vacancy Ordering in GeSbTe Phase Change Materials

**DOI:** 10.1038/srep23843

**Published:** 2016-04-01

**Authors:** Valeria Bragaglia, Fabrizio Arciprete, Wei Zhang, Antonio Massimiliano Mio, Eugenio Zallo, Karthick Perumal, Alessandro Giussani, Stefano Cecchi, Jos Emiel Boschker, Henning Riechert, Stefania Privitera, Emanuele Rimini, Riccardo Mazzarello, Raffaella Calarco

**Affiliations:** 1 Paul-Drude-Institut für Festkörperelektronik, Hausvogteiplatz 5-7, 10117 Berlin, Germany; 2 Dipartimento di Fisica, Università di Roma “Tor Vergata”, Via della Ricerca Scientifica 1, I-00133 Rome, Italy; 3 Institut für Theoretische Festkörperphysik and JARA, RWTH Aachen University, D-52056 Aachen, Germany; 4 Center for Advancing Materials Performance from the Nanoscale, State Key Laboratory for Mechanical Behavior of Materials, Xi’an Jiaotong University, Xi’an 710049, PR China; 5 Institute for Microelectronics and Microsystems (IMM), Consiglio Nazionale delle Ricerche (CNR), VIII Strada, 5-95121 Catania, Italy

## Abstract

Phase Change Materials (PCMs) are unique compounds employed in non-volatile random access memory thanks to the rapid and reversible transformation between the amorphous and crystalline state that display large differences in electrical and optical properties. In addition to the amorphous-to-crystalline transition, experimental results on polycrystalline GeSbTe alloys (GST) films evidenced a Metal-Insulator Transition (MIT) attributed to disorder in the crystalline phase. Here we report on a fundamental advance in the fabrication of GST with out-of-plane stacking of ordered vacancy layers by means of three distinct methods: Molecular Beam Epitaxy, thermal annealing and application of femtosecond laser pulses. We assess the degree of vacancy ordering and explicitly correlate it with the MIT. We further tune the ordering in a controlled fashion attaining a large range of resistivity. Employing ordered GST might allow the realization of cells with larger programming windows.

Phase change materials (PCMs) are unique compounds that find their application in electrical and optical storage media^1^ due to the large differences in optical and electrical properties between their amorphous and crystalline phases.

Amongst different compounds exhibiting PCM properties the most interesting are Te-based alloys, typified by Ge-Sb-Te (GST), as they exhibit the ability to switch between phases on the nanosecond time scale. Especially Ge_2_Sb_2_Te_5_ (GST225) can be considered prototypical. Amorphous thin films, grown by techniques such as sputtering, crystallize into a metastable rocksalt structure^2^, although the stable configuration of the crystalline phase of GST belongs to the trigonal crystal system with rhombohedral space group (P
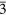
m1 or R
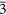
m)^3^. In the current study, in line with literature^4^, we define for simplicity the metastable phase as cubic, and the stable phase as hexagonal.

GST contains a large amount of vacancies as a function of alloy composition^5^. Ab-initio calculations have suggested that spatial separation and ordering of Ge, Sb, and vacancies lower the total energy of the system^6^,^7^. Further density functional theory (DFT) calculations confirmed that different metastable cubic and hexagonal phases can be achieved depending on the ordering of the vacancy distribution^8^. The difference between the ordered cubic structures and the hexagonal phases is the stacking of the layers (with reference to the Te sublattice), which means *ABCABC* for cubic and *ABCBCA* for hexagonal GST225^6^.

Recently, experimental results on polycrystalline GST (poly-GST) films evidenced a metal-insulator-transition (MIT)^9^, which was attributed to disorder (Anderson MIT). Subsequently, *ab initio* simulations indicated that the insulating behaviour stems from the localization of electronic states around vacancy clusters, whereas the transition to the metallic state is induced by the ordering of vacancies into layers^8^. So far, only poly-GST has been investigated and no experimental evidence of the influence of vacancy layers on the MIT has been given. An important breakthrough would be to correlate experimentally the resistivity change with the vacancy ordering. For this purpose, the challenge is to fabricate highly ordered single-crystalline material that facilitates the direct experimental observation of the vacancy layers. In addition, such material will make it possible to control the MIT transition by finely tuning the disorder in the material.

Growth of epitaxial GST alloys via molecular beam epitaxy (MBE) has recently been demonstrated and carried out on different substrates and orientations to obtain films with a single out-of-plane crystalline orientation^10^,^11^.

Here we present a fundamental advance in the fabrication of PCMs through MBE resulting in as-deposited single-crystalline GST with out-of-plane stacking of vacancy layers (VLs). Single-crystalline, highly-ordered GST is also obtained-through annealing treatment and via fs pulse laser crystallization of amorphous GST (a-GST) deposited on a crystalline substrate, which acts as a template for the crystallization. Most interestingly, the possibility to tune the degree of vacancy ordering, combined with low temperature transport measurements, allows us to correlate the ordering degree in GST with the MIT.

## Results and Discussion

**Figure 1:**
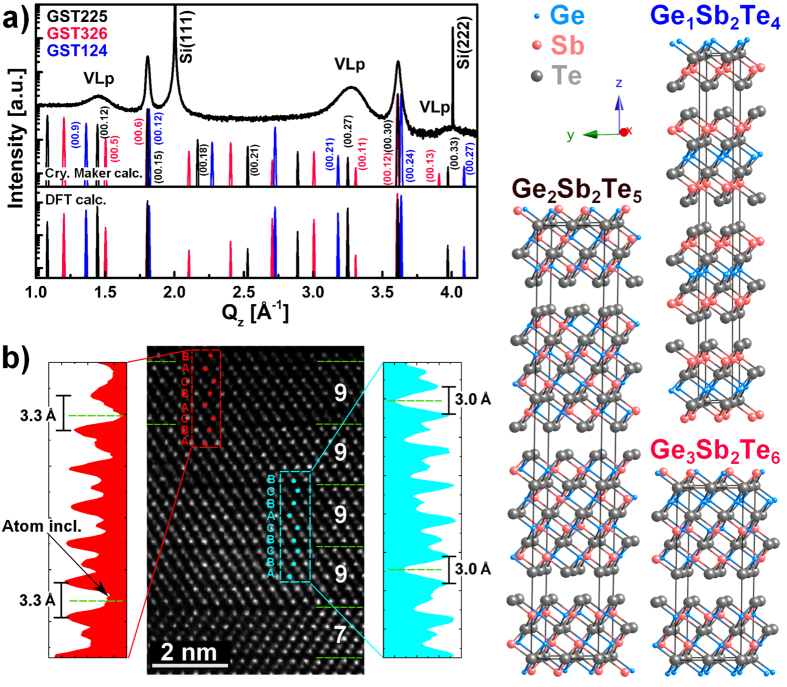
(**a**) Superimposed ω-2ϑ scans for crystalline GST grown by MBE (black curve) and simulations of the GST225 (326) [124] in black (red) [blue] performed by using Crystal Maker®^12^ and DFT calculations. The corresponding crystal structures are displayed in the right panel. (**b**) Cross-view [11.0] high resolution HAADF STEM micrograph of epitaxial (00.1) GST; VLs (dashed green lines) occur every 7–9 atomic layers. Cubic (red) and rhombohedral stacking (light blue) in respect to the Te sublattice is highlighted and corresponding integrated line profiles along the [00.1] direction are shown.

We first focus on the structural characteristics of highly textured GST with optimized stacking. In Fig. 1(a) we present the XRD profile (symmetric ω-2ϑ scans along the [00.1] direction) of a GST sample. Peaks at Q_z_ = 2.00, 4.01 Å^−1^ are attributed to the Si substrate while two narrow (Q_z_ = 1.81, 3.61 Å^−1^) peaks are ascribed to the GST epilayer. The latter are multiple order Bragg reflections of the GST epilayer corresponding to the (00.15) and (00.30) planes respectively. Note that we describe the GST unit cell with hexagonal axes, for more details see Supplementary Information (SI)-XRD simulations. Interestingly, three additional broader peaks (Q_z_ = 1.44, 3.27, 4.00 Å^−1^) appear, which are not Bragg reflections of the GST unit cell (not integer order reflections of the first peak). Analysis of peak position and intensities excludes phase separation, atomic segregation or other crystallographic orientations. The effect of strain is not the cause of such peaks either (see SI Figure S1). GST was also grown on almost lattice matched InAs with (001) and (111) orientations to discard effects related to mismatch and crystal orientation (see SI Figure S2). Therefore, the appearance of such peaks indicates the presence of occupational modulation of the individual Ge, Sb atoms or vacancies in the Ge/Sb sublattice, for instance, along the [00.1] direction.

To clarify this point, we carried out a series of simulations of the XRD data by using the Cystal Maker® and Crystal Diffract® software packages^12^. Symmetric ω-2ϑ scans along the [00.1] direction were calculated. Starting from the cubic phase, several model structures were considered, differing in the distribution and ordering of the vacancies (see SI for more details). Figure 1(a) shows the simulated XRD profile for the model structures displayed on the right side of the panel, where perfectly ordered distributions of vacancies were introduced to simulate the formation of fully depleted VLs for the phases 225, 326 and 124 of GST, as in the structures proposed by Da Silva *et al*.^6^. The VLs generate a superstructure from which additional maxima in the simulated XRD profile emerge, henceforth called vacancy layer peaks (VLp). The reflections at Q_z_ = 1.44, 3.27, and 4.00 Å^−1^ in the GST experimental profile (black curve in Fig. 1(a)), can be identified as the reflections (00.12), (00.27) and (00.33) in hexagonal notation, respectively, of GST225; they can be viewed as the first order satellite peaks of the VL superstructure. By filling the VLs with Sb and Ge atoms, the VLps’ intensity in the simulated profile were reduced and disappeared when the concentration of vacancies for each plane was less than 25%.

The full width at half maximum (FWHM) of the experimental VLps is very large if compared to the basic GST peak ((00.15) and (00.30) reflections). The observed differences between the simulations (ideal crystals) and the experimental results can be explained if we assume a certain statistical disorder in the layer sequence which includes a distribution of stacking faults (variable number of planes in between the VLs-see SI) and a random distribution of vacancies among the planes. The stacking sequence of atomic (00.1) GST layers and the VLs are the main features that directly affect the symmetric XRD along the [00.1] direction. On the other hand, the presence of stacking faults and fluctuations in the vacancies occupation can be viewed as the coexistence of GST phases other than 225 (see the structures in Fig. 1). The first order satellite peaks of the various phases (225, 326, 124) are very close to each other, explaining the observed FWHM of the VLPs as well as the strong intensity reduction of the higher order satellite peaks.

To take into account the relaxation of atoms at the VLs, DFT calculations were performed on the same GST structures of Fig. 1 (see SI). The two simulations [lower panel of Fig. 1(a)] agree very well in terms of the peaks position while they differ in the intensity (in particular of the (00.18) and (00.21) reflections).

By VLs we identify depleted Sb/Ge layers without any structural rearrangement, but, in accordance with Zhang *et al*.^8^, the Te-Te distance at the two sides of a VL decreases significantly with increasing degree of ordering (depletion degree), till van der Waals (vdW) gaps are formed in the most ordered hexagonal phase. For simplicity, we continue referring to them as VLs. A clear example of stacking faults and mixed phases is evident in Fig. 1(b) where a high-angle annular dark field scanning transmission electron microscopy (HAADF-STEM) image of a GST sample is presented. The film consists of domains with a (00.1) out-of-plane orientation with evident intensity modulations (dark lines), consistent with the presence of stacking faults associated with VLs and is observed every 7 to 11 atomic planes [Fig. 1(b)]. For GST225, 9 atomic planes are expected to form between two subsequent VLs, 11 for GST326 and 7 for GST124 (see Fig. 1). From the intensity modulation in the profile the layers occupied by Te are clearly evidenced, while the intensity occupied by cations, indicates a mixed Sb/Ge occupancy. The STEM data corroborate the conclusions that our highly ordered samples are constituted by a GST alloy with 225 average composition reconciled with the 124 and 326 phases. Furthermore, both cubic and rhombohedral stacking can be identified (red and light blue boxes, respectively, in Fig. 1(b)). Both stacking coexist across the VLs and show a different degree of Sb/Ge depletion: atomic inclusions are visible in the gap in case of the cubic stacking. Te-Te layer distance upon the gap differs for the cubic (3.3 Å) and hexagonal (3.0 Å) case, in accordance with Zhang *et al*.^8^. This indicates that the cubic to hexagonal transition is a continuous process where both cubic and rhombohedral stacking coexist with a high degree of ordering. We thus demonstrated that a highly ordered GST crystalline phase with vacancies arranged on (00.1) planes is achieved by MBE growth, which was so far only theoretically predicted^6^,^8^. It is now of high interest to investigate whether ordered GST can be obtained by thermal annealing that delivers a simple way to control the resistivity of the produced film^9^. From previous investigations we have shown that a crystalline Si (111) substrate acts as a template for the crystallization via annealing of GST films, since they share the same out-of-plane orientation: [111]Si || [00.1]GST^13^. This feature was also reported for fs laser amorphized and recrystallized GST, epitaxially grown on crystalline substrates^14^. We therefore deposited an a-GST film on a Si (111) substrate and investigated the crystalline phase obtained by annealing above the crystallization temperature.

**Figure 2:**
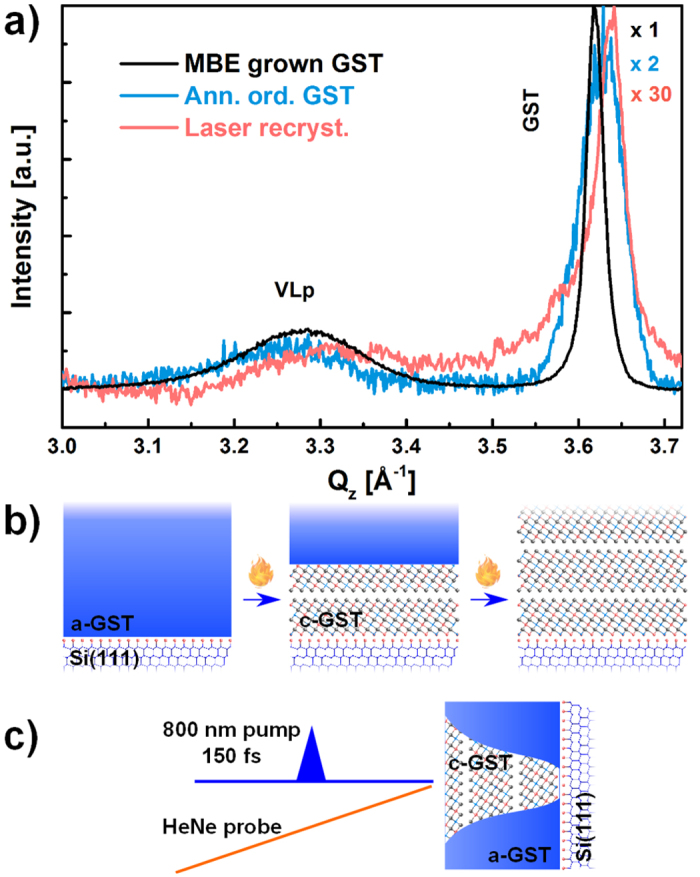
(**a**) Superimposed ω-2ϑ scans for crystalline GST on Si (111) grown by MBE (black), a-GST crystallized by isothermal annealing (light blue) and by fs laser pulses (light red). All the curves are normalized to the GST peak and the multiplication factors are reported on the side. (**b**) Schematic of the crystallization process of a-GST deposited on Si (111) by annealing and (**c**) by application of fs laser pulses.

In Fig. 2(a) ω-2ϑ scans around the GST (00.30) peak for a crystalline MBE grown sample (black) and for a sample crystallized by means of an isothermal annealing at 120 °C for 20 hours (light blue) are compared. The peaks at Q_z_ = 3.27 Å^−1^ (black curve) and Q_z_ = 3.26 Å^−1^ (light blue curve) are attributed to the VLp. This demonstrates that a highly ordered GST with vacancies arranged in the (00.1) planes is achieved also by means of thermal annealing of MBE deposited a-GST, as schematically shown in Fig. 2(b).

Thermal annealing is a slow process, while the switching in PCM devices occurs on a time scale of about 100 ns. In order to achieve faster crystallization, we intend to obtain ordered GST by crystallization of a-GST with short pulses. Laser radiation of 800 nm with 150 fs long pulses is used for recrystallization of a-GST and a He-Ne laser allowed the change of reflectivity from amorphous to crystalline phase to be detected (see Fig. 2(c)). In this case high texture and a faint vacancies ordering are obtained [Fig. 2(a) (light red)]. The tiny difference in GST(00.30) peak position after both recrystallization experiments is due to slight changes in composition. The samples were intentionally left uncapped to avoid strain and heterogeneous nucleation sites and thus a preferential desorption may occur^13^. The overall lower intensity of the peaks in the laser annealed sample is attributed to a smaller percentage of crystallized volume, and to laser induced local damage of the film. Note that the crystallization into ordered crystalline phase may proceed via a two-step process, involving the initial formation of a disordered cubic phase and the subsequent ordering of the vacancies, as indicated by the *ab initio* molecular dynamics simulations of crystallization of GST in the presence of a two dimensional crystalline template^15^.

These results unequivocally show that vacancy ordering is achieved by crystallization of an a-GST film deposited on a crystalline substrate by either thermal annealing or application of short laser pulses. This finding is remarkable as it demonstrates that it is possible to create an ordered crystalline GST layer starting from a-GST and using different fabrication procedures.

Ordering of vacancies is therefore expected also in poly-GST.

**Figure 3:**
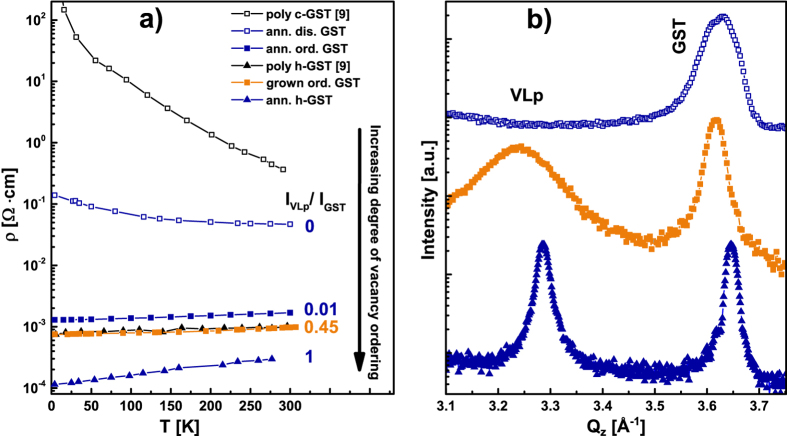
(**a**) Resistivity as a function of temperature for crystalline GST samples (blue and orange) compared with poly-GST (black)^9^. Empty and filled symbols denote disordered and ordered GST, while triangles hexagonal GST. Intensity ratio (I_VLp_/I_GST_) of VLp and GST peak is reported for the epitaxial samples. (**b**) ω-2ϑ scans around the VLp and GST peak for different degrees of ordering.

The ordering of vacancies into VLs and later vdW gaps is expected to provide a reduction in the film resistivity. To this end, in Fig. 3(a) we compare the in-plane resistivity as a function of temperature for different samples. a-GST annealed at 110 °C for 10 min (empty blue squares), which is crystalline but not yet ordered in term of vacancy planes, shows a negative temperature coefficient slope indication of a non metallic behaviour. Instead, the GST annealed at 170 °C for 1 h (filled blue squares), the as grown highly ordered GST with both cubic and rhombohedral stacking (filled orange squares), the GST in hexagonal phase obtained by annealing at 270 °C for 1 h (filled blue triangles) all show a positive temperature coefficient slope (metallic behaviour) with systematically decreasing resistivity. This indicates a progressive increase in vacancy ordering, as given by the XRD intensity ratio (I_VLp_/I_GST_) between the VLp and the GST (00.30) peak. I_VLp_/I_GST_ ranges from zero (empty blue squares) for the disordered GST up to 1 (filled blue triangles) for the hexagonal GST. As already pointed out by Siegrist *et al*.^9^, the MIT in GST is not due to a cubic to hexagonal phase transition but, as proposed by Zhang *et al*.^8^, it relates to the ordering of localized vacancy clusters into layers. For comparison measurements taken from Siegrist *et al*.^9^ relative to cubic poly-GST (empty black squares) and hexagonal GST (filled black triangles) are shown. It is evident that the resistivity values for poly-GST are systematically higher as compared to the highly ordered single-crystalline counterpart. In particular, highly-ordered as-grown GST shows resistivity similar to the hexagonal poly-GST. We thus enlarge the range of resistivity by employing epitaxially grown GST. Most importantly, the present study definitely establishes a direct link between ordering in GST and the MIT and thus provides the experimental confirmation of theoretical prediction^8^.

In addition we show that the MIT occurs within the onset of cubic phase formation (non ordered annealed at 110 °C for 10 min) and the slightly ordered cubic phase, as seen by the sample annealed at 170 °C for 1 h that displays a metallic behaviour and a I_VLp_/I_GST_ = 0.01.

In conclusion we have demonstrated that it is possible to fabricate single-crystalline and highly ordered GST, which was so far only predicted by theoretical calculations^6^,^7^. The ordering of vacancies into layers was obtained for both MBE as grown crystalline GST, and a-GST crystallized by conventional annealing or fs-laser pulses. This indicates that the ordering in GST occurs regardless of the fabrication method. In particular, the fabrication of single-crystalline material facilitates the direct observation of the VLs via XRD and TEM and allows us to clearly demonstrate that the MIT in GST starts in the cubic phase and arises from ordering, where the formation of VLs plays a fundamental role. Most interestingly, the degree of ordering can be tuned in a controlled fashion, enabling us to obtain a large range of resistivity. In particular, this finding is promising for the realization of memory cells. Very recently the MBE technique was successfully used to fabricate poly-GST in memory cells resulting in state of the art electrical performance^16^. Employing ordered GST might offer benefits similar to chalcogenide superlattices^17^ and allow the realization of cells with larger programming windows.

## Methods

### MBE growth

Growth of epitaxial GST on Si (111) substrates was performed employing molecular beam epitaxy. Prior deposition, the Si substrates (p-type) with resistance of 1–10 Ω*cm, were prepared using standard procedure^11^,^18^ and introduced in the MBE system for backing procedure as described elsewhere^19^ before being introduced in the growth chamber. Here the Sb-terminated Si (111) surface reconstruction is obtained by different annealing (see Boschker *et al*.^20^ for details). Surface reconstructions and *in-situ* deposition characterization are performed by reflection high-energy electron diffraction. The deposition of the GeTe-Sb_2_Te_3_ alloys was performed at substrate temperature of 250 °C in a vacuum condition of ~10^−10^ mbar. Flux ratio between the elemental sources of the GST where properly chosen in order to obtain layers with different compositions. In the specific case of the GST225 composition, flux ratios of 3.2 and 2.1 were used for Sb/Ge and Te/Sb.

### XRD

Samples were characterized by means of *ex-situ* X-ray diffraction (XRD), utilizing a PANalytical X’ Pert PRO MRD diffractometer with Ge (220) hybrid monocromator, Employing a Cu Kα_1_ radiation (λ = 1.540598 Å). Specular ω-2ϑ scans were performed in double axes mode in order to access the growth direction of the films, in a range of 10°–110°, with a step 0.02° and integration time of 2.5 s.

### HAADF-STEM

JEOL ARM200F Cold FEG STEM working at 200 kV was adopted to obtain High Resolution micrographs of the sample. The High Angle Annular Dark Field STEM images were obtained with a convergence semiangle of 33 mrad, allowing for a nominal resolution of about 0.68 Å, with an inner detection semiangle of 83 mrad. Scanning was performed using a dwell time per pixel of 40 μs and a beam current of ≈50 pA. The film was observed in the [110] zone axis, containing the c-axis direction, in order to directly observe the stacking sequence of the atomic planes along it. HAADF-STEM directly relates the micrograph contrast to the atomic number (Z-contrast), allowing a straightforward interpretation of the images in terms of Ge/Sb and Te layers, plane spacing and van der Waals gap presence. The sample was prepared by standard cross-sectional mechanical polishing followed by Ar+ ion milling at LN_2_ temperature, using a Gatan PIPSII system. Ion energy was set from 2.0 keV to 0.1 keV in order to avoid sample amorphization and damaging.

Average composition is measured by energy dispersive X-ray diffraction.

### Laser crystallization of a-GST

The crystallization experiment of a-GST layers through laser radiation was performed employing as a pump, a 800 nm Ti:Sa pulsed laser with pulse duration of 150 fs and temporal Gaussian pulse shape, repetition rate of 1 kHz, which reduces the energy loss due the heat transfer in the sample. In order to monitor *in-situ* the change in reflectivity upon amorphous to crystalline phase transition, a He-Ne laser, overlapped to the pump, was used as a probe (see schematic in Fig. 2(c)). The thickness of the a-GST sample was chosen in such a way to match the penetration depth of the laser, which can reach the Si substrate, the latter important as template for the crystallization^13^,^14^ (Fig. 2(c)).

### Thermal annealing

Rapid thermal annealing of a-GST was performed under 1 bar nitrogen atmosphere. Further details can be found elsewhere^13^.

### Electrical measurements

Lateral electrical transport properties of the GST samples were studied employing a temperature dependent Hall measurements setup. GST samples with thicknesses of ~30 nm were cut in square shapes of 5 × 5 mm^2^. In balls and Au bonds were used for the contacts in order to perform low temperature measurements (4–300 K) in a four-contact van der Pauw configuration. For the measurements, an electrical and magnetic field of 10 mA and 0.25 T were applied, respectively.

## Additional Information

**How to cite this article**: Bragaglia, V. *et al*. Metal - Insulator Transition Driven by Vacancy Ordering in GeSbTe Phase Change Materials. *Sci. Rep.* **6**, 23843; doi: 10.1038/srep23843 (2016).

## Supplementary Material

Supplementary Information
