# Corrigendum: Metal - Insulator Transition Driven by Vacancy Ordering in GeSbTe Phase Change Materials

**DOI:** 10.1038/srep31679

**Published:** 2016-08-16

**Authors:** Valeria Bragaglia, Fabrizio Arciprete, Wei Zhang, Antonio Massimiliano Mio, Eugenio Zallo, Karthick Perumal, Alessandro Giussani, Stefano Cecchi, Jos Emiel Boschker, Henning Riechert, Stefania Privitera, Emanuele Rimini, Riccardo Mazzarello, Raffaella Calarco

Scientific Reports 6: Article number: 2384310.1038/srep23843; published online: 04 01 2016; updated: 08 16 2016

In this Article, Stefania Privitera and Emanuele Rimini are incorrectly listed as being affiliated with ‘Paul-Drude-Institut für Festkörperelektronik, Hausvogteiplatz 5-7, 10117 Berlin, Germany’. The correct affiliation is listed below:

Institute for Microelectronics and Microsystems (IMM), Consiglio Nazionale delle Ricerche (CNR), VIII Strada, 5-95121 Catania, Italy.

