# Supplementary material for: Metal - Insulator Transition Driven by Vacancy Ordering in GeSbTe Phase Change Materials
Source: Sci Rep. 2016 Apr 1;6:23843. doi: 10.1038/srep23843 (PMC4817142; doi:10.1038/srep23843)
Supplement: Supplementary Information [file srep23843-s1.pdf]

Supplementary Information

Metal - Insulator Transition Driven by Vacancy Ordering in GeSbTe Phase Change Materials

*Valeria Bragaglia[1], Fabrizio Arciprete[1,2], Wei Zhang[3,4], Antonio Massimiliano Mio[5], Eugenio Zallo[1], Karthick Perumal[1], Alessandro Giussani[1], Stefano Cecchi[1], Jos Emil Boschker[1], Henning Riechert[1], Stefania Privitera[1], Emanuele Rimini[1], Riccardo Mazzarello[3], Raffaella Calarco[1]**

[1]Paul-Drude-Institut für Festkörperelektronik
Hausvogteiplatz 5-7, 10117 Berlin, Germany

[2]Dipartimento di Fisica, Università di Roma "Tor Vergata",
Via della Ricerca Scientifica 1, I-00133 Rome, Italy

[3]Institut für Theoretische Festkörperphysik and JARA, RWTH Aachen University,
D-52056 Aachen, Germany

[4]Center for Advancing Materials Performance from the Nanoscale, State Key Laboratory for Mechanical Behavior of Materials, Xi'an Jiaotong University, Xi'an 710049, PR China

[5]Institute for Microelectronics and Microsystems (IMM), Consiglio Nazionale delle Ricerche (CNR),VIII Strada, 5 - 95121 Catania, Italy

XRD characterization

In Figure S1 a reciprocal space map (RSM) around the asymmetric Si (224) Bragg peak in coplanar configuration is shown. The axes are reported in reciprocal space units with $Q_z \parallel$ Si[111] and $Q_x \parallel$ Si[-1-12]. From the coordinates of the GST (01.13) peaks it is possible to extract both the in-plane ($a_{\parallel}$) and out-of-plane ($a_{\perp}$) lattice constants of the GST epylayer. In particular $a_{\parallel}$ is 4.24 Å while $a_{\perp}$ is 3.53 Å, which corresponds to the distance of a bilayer of GST in the [00.1] direction. The values compare fairly good with the theoretical data reported by Da Silva et al.[1]. From the relative distance between the Si (224) and the GST peak it is possible to estimate the degree of relaxation of the film. In fact the black line in Figure S1 represents the pseudomorphic case, while the magenta line was drawn using a literature

reference for totally relaxed GST film (lattice constant $a_{||}$ = 4.26 Å)[2]. The grown GST film clearly matches the magenta line and this indicates that it grows relaxed.

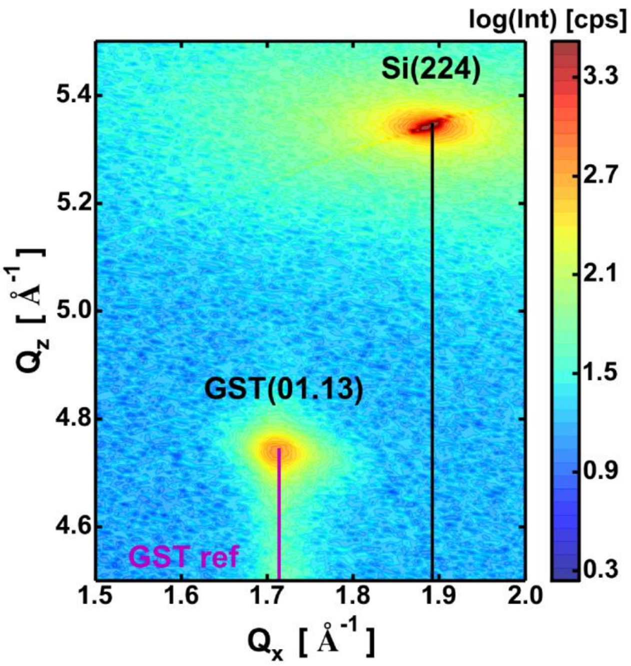

Figure S1: RSM around the asymmetric Si (224) Bragg peak with Q_z || Si[111] and Q_x || Si[-1-12]. The intensity is given in arbitrary units according to the logarithmic scale on the side

Out of plane XRD of GST grown on substrates with crystallographic orientations different than the [111] does not show vacancy layer peaks (VLps). In particular in Figure S2 a comparison of symmetric ω-2ϑ XRD scans for GST grown on InAs (001) and (111) substrates is shown. GST and InAs present a very low lattice mismatch ~ 0.77 % if compared with the Si substrate (~ 9.4 %), for this reason in the XRD profiles the peaks corresponding to the substrate and to the GST almost superimpose. For the GST grown on the InAs (001) (black curve) we can identify two sharp peaks at Q_z = 2.04 and 4.13 Å^{-1}, which are attributed to the substrate first and second order of the [001] orientation, and two resolved peaks with slightly higher scattering angles at Q_z = 2.08 and 4.17 Å^{-1}. They are attributed to the GST (20.5) and (40.10) Bragg reflections respectively. The small bump at Q_z = 3.58 Å^{-1} which is ~ 3 order of magnitude smaller than the main reflection is attributed to GST (00.30)

as already reported[3,4]. In the blue curve corresponding to the GST grown on the InAs (111), the GST and substrate peaks are less resolved. However, similarly to the case of GST grown on Si (111), two additional features are visible at Q_z = 1.61 and 3.41 $Å^{-1}$. As no correspondence with any crystallographic orientation other than the [00.1] was found, we than conclude that these reflections are VLps.

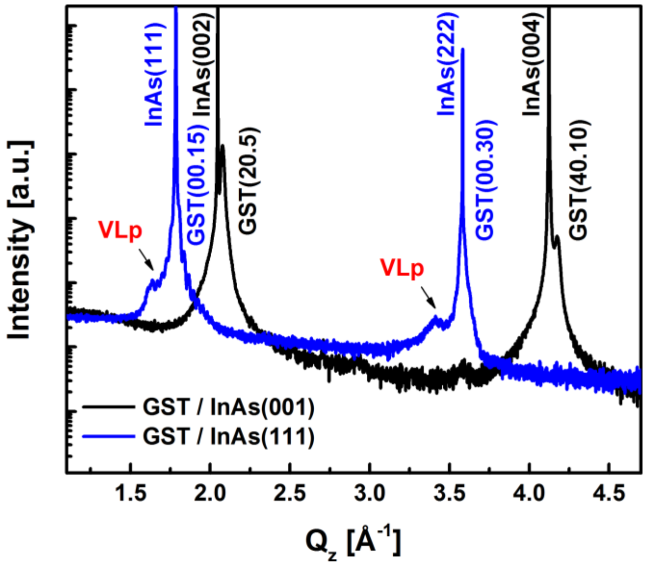

Figure S2: Symmetric ω-2ϑ XRD scans for GST grown on InAs (001) (black curve) and InAs (111) (blue curve)

XRD simulations

From the GST (111), (222), (333) peaks of our XRD spectra we can determine the lattice parameter along the c axis, this giving 17.39 Å (the building block in between vacancy layers (VLs) see the model structure in Figure 1 of the main text). Such value agrees perfectly with the experimental value given in literature for the metastable 225 phase[5]. Therefore, in order to simulate our XRD spectra for the 225, 326 and 124 phases of GST, by using the Crystal Maker[6] software package, several metastable cubic phases, depending on the distribution and ordering of the vacancies, were considered. The first considered structure was the metastable rocksalt cubic phase for the GST225 (space group Fm-3m), where Sb, Ge and vacancies are randomly distributed in the cation sublattice. The average occupancy for Ge and Sb is 0.392

while the vacancy concentration is 21.6 %. The simulated XRD spectrum exhibits the three Bragg reflections (111), (222), and (333) and no other additional peaks. In Figure S3(a) the simulation is reported in the Q_z region around the GST (222)-peak after a fine tuning of the GST cubic lattice parameter to fit the experiment, this giving a = 6.0108 Å. The next step consisted of introducing a more ordered distribution of vacancies, simulating the formation of VLs. If we consider the cubic structure along the [111] direction, the vacancy planes can be achieved by moving the vacancies (v) from every Sb/Ge/v plane to each fifth Sb/Ge/v plane until it is a complete vacancy plane. In Figure S3(b) the new structure is represented in the conventional hexagonal lattice (the unit cell containing 3 f.u.) where we can observe a sequence of Te and Sb/Ge/v planes along the [00.1] direction with a VL every four Sb/Ge layers (VL model 1). We can consider the new structure a superlattice of Sb_2Te_3 and GeTe in which the stacking sequence is still *ABCABC* as in the rocksalt structure. The simulated XRD is reported in Figure S3(a). The diffraction maxima are now indexed as (00.n) according to the hexagonal unit cell reported in Figure S3(b). The XRD maximum (00.30) coincides with the (222) peak of the cubic rocksalt structure. However, the simulation's most important result is the appearance of a series of new maxima with n = 3p (p is an integer), i.e. only diffraction peaks with n equal to a multiple of three appear. This is due to the fact that the unit cell is composed of three building blocks, stacked along the [00.1] direction and separated by the VLs, which are identical in the symmetric diffraction geometry. The presence of the vacancy planes gives rise to a superstructure from which additional maxima in the simulated XRD arise. As in the cubic structure, the lattice parameter c of the model structure was adjusted to fit the (00.30) reflection of the simulated spectrum to the experimental peak, this producing c = 52.1965 Å. The reflections at 3.27 and 4.00 $Å^{-1}$ in the experimental spectrum can be identified as the reflections (00.27) and (00.33) in hexagonal notation, respectively; they can also be viewed as the satellite peaks of the superstructure containing the vacancy planes. Furthermore, the full width at half maximum (FWHM) of the VLps is very large if compared

to the basic GST peak ((00.30) reflection). To understand this point we simulated a new structure where we introduced a stacking fault of the vacancy layers by exchanging one of the vacancy planes with one of the adjacent Sb/Ge planes. In such a model a new periodicity along the c axis is introduced and the selection rule allowing only the (00.3p) reflections is removed. The new simulated spectrum (VL model 1 SF in Figure S3(a)) exhibits two equally spaced peaks in between the peaks of the unfaulted structure. The model structures in Figure S3(b) represent ideal crystals while the simulations in Figure S3(a) represent the XRD from ideally ordered phases. Therefore, the observed differences between the simulations and the experimental results can be explained if we assume a certain statistical disorder in the layer sequence which includes a distribution of stacking faults. This explains the observed FWHM of the VLps as well as the strong reduction of the higher order satellite peaks (see main text).

In previous models we only considered an average occupation for Sb and Ge atoms in the planes in between the Te layers (with the obvious exception of the vacancy planes). This does not affect significantly the simulation of symmetric XRD along the [00.1] direction. As an example of this aspect, we modelled a structure (see Figure 1 of the main text) for the GST225 where the vacancy planes are fully depleted, the Sb/Ge planes are fully occupied and each Sb(Ge) atom is coordinated by four Ge(Sb) atoms, as in the model proposed by Da Silva et al.[1]. The simulated XRD is shown in Figure S3(a) (VL model 2); no significant difference can be observed with respect to the VL model 1, with the exception of only tiny differences in the intensity of some reflections.

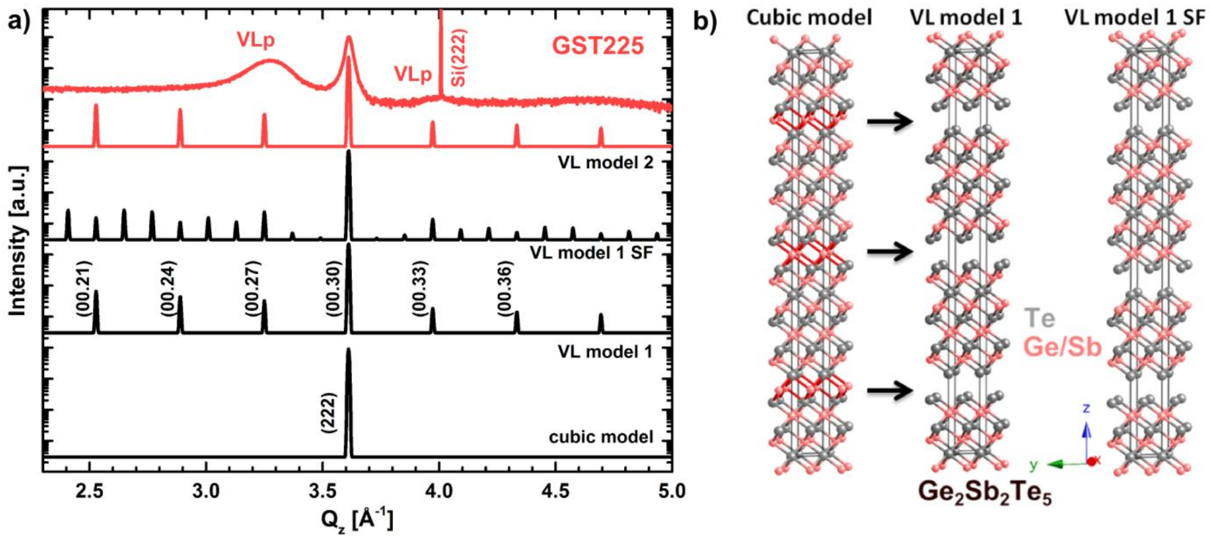

Figure S3: a) Experimental ω -2ϑ scan of the second order for crystalline GST225. The simulations performed by Crystal Maker are based on different models, as reported (see labels). b) Corresponding crystal structures for the several VL models employed.

DFT

Density functional theory simulations were performed using the code PWSCF included in the Quantum Espresso package[7]. Ultrasoft pseudopotentials[8,9], and PBE exchange-correlation functionals[10] were employed. Van der Waals corrections are included by using the Grimme's D2 method[11]. An energy cutoff of 480 eV was employed. The Brillouin zone was sampled with a Monkhorst-Pack (MP) mesh[12]. The atomic positions in all the models were fully relaxed. Experimental lattice constants were used, namely a = 4.26 Å, c = 20.9 Å ($Ge_3Sb_2Te_6$); a = 4.26 Å, c = 52.2 Å ($Ge_2Sb_2Te_5$); a = 4.26 Å, c = 41.5 Å ($Ge_1Sb_2Te_4$).

Orthorombic unit cells ($\sqrt{3} \times 1 \times 1$) were considered for ordered $Ge_3Sb_2Te_6$, $Ge_2Sb_2Te_5$ and $Ge_1Sb_2Te_4$ with cubic stacking. The corresponding stacking sequences were Te-Sb-Te-Ge-Te-Ge-Te-Ge-Te-Sb-Te, Te-Sb-Te-Ge-Te-Ge-Te-Sb-Te (× 3) and Te-Sb-Te-Ge-Te-Sb-Te (× 3), respectively. Note that the difference between these ordered structures and the corresponding hexagonal phases is the relative position between the three adjacent building blocks[1]. X-Ray Diffraction simulations were performed based on the relaxed structures with Crystal Maker[6].

In order to determine the effects of compositional disorder on XRD patterns, orthorombic super cells ($2\sqrt{3} \times 1 \times 1$) were built and relaxed for $Ge_3Sb_2Te_6$, $Ge_2Sb_2Te_5$ with the stacking sequence Te-Sb/Ge-Te-Sb/Ge-Te-Ge-Te-Sb/Ge-Te-Sb/Ge-Te, Te-Sb/Ge-Te-Sb/Ge-Te-Sb/Ge-Te-Sb/Ge-Te, respectively. The mixed layers were occupied by 50 % Ge and 50 % Sb atoms. In these supercells, 12 Ge/Sb atoms were randomly distributed on each mixed atomic layer. The simulated XRD patterns of the clean and disordered models differ slightly in the intensity of the peaks only, whereas the positions of the peaks are identical. The employed k point mesh was $4 \times 6 \times 1$ for the clean $Ge_2Sb_2Te_5$ and $Ge_1Sb_2Te_4$, and $4 \times 6 \times 2$ for the clean $Ge_3Sb_2Te_6$, and gamma point for the disordered $Ge_2Sb_2Te_5$ and $Ge_3Sb_2Te_6$.

Laser crystallization of a-GST

The crystallization experiment of a-GST layers through laser radiation was performed with a fluence of 14 mJ/cm^2 in order to maximize the relative reflectivity change upon phase transition (~ 10 %). Due to the X-ray beam detection size (1×10 mm^2), a matrix of adjacent laser induced crystalline spots of 400 µm in diameter were written on the a-GST. For each spot of the matrix, a sequence of fs pulses was delivered and the change of reflectivity as a function of time was monitored by an oscilloscope connected to the probe laser. Considering the pump repetition rate and the time it takes to reach the total relative reflectivity change (300 ms), it was estimated that the needed number of fs pulses for crystallization is ~ 300. In addition, if we consider the time separation of 1 ms between two fs pulses, it is reasonable to assume that the crystallization process is faster than 300 ms with a lower limit of 45 ps.

REFERENCES

1. Da Silva, J., Walsh, A. & Lee, H. Insights into the structure of the stable and metastable (GeTe)m(Sb2Te3)n compounds. *Phys. Rev. B* **78,** 224111 (2008).

2. Matsunaga, T., Yamada, N. & Kubota, Y. Structures of stable and metastable Ge2Sb2Te5, an intermetallic compound in GeTe-Sb2Te3 pseudobinary systems. *Acta Crystallogr. B.* **60,** 685–91 (2004).

3. Braun, W. Epitaxy of phase change materials. in *Spring MRS 2010* 1–32 (2010).

4. Shayduk, R. & Braun, W. Epitaxial films for Ge-Sb-Te phase change memory. *J. Cryst. Growth* **311,** 2215–2219 (2009).

5. Matsunaga, T. *et al.* Single structure widely distributed in a GeTe-Sb2Te3 pseudobinary system: A rock salt structure is retained by intrinsically containing an enormous number of vacancies within its crystal. *Inorg. Chem.* **45,** 2235–2241 (2006).

6. CrystalMaker. Software Oxford, United Kingdom. at <www.crystalmaker.com>

7. Giannozzi, P. *et al.* QUANTUM ESPRESSO: a modular and open-source software project for quantum simulations of materials. *J. Phys. Condens. Matter* **21,** 395502 (2009).

8. Vanderbilt, D. Soft self-consistent pseudopotentials in a generalized eigenvalue formalism. *Phys. Rev. B* **41,** 7892–7895 (1990).

9. Dal Corso, A. Pseudopotentials periodic table: From H to Pu. *Comput. Mater. Sci.* **95,** 337–350 (2014).

10. Perdew, J. P., Burke, K. & Ernzerhof, M. Generalized Gradient Approximation Made Simple. *Phys. Rev. Lett.* **77,** 3865–3868 (1996).

11. Grimme, S. Semiempirical GGA-type density functional constructed with a long-range dispersion correction. *J. Comput. Chem.* **27,** 1787–1799 (2006).

12. Monkhorst, H. J. & Pack, J. D. Special points for Brillouin-zon integrations. *Phys. Rev. B* **13,** 5188–5192 (1976).
